# Rapid progress towards elimination of lymphatic filariasis in endemic regions of Myanmar as a result of 16 years of anti-filarial activities (2001–2016)

**DOI:** 10.1186/s41182-018-0093-x

**Published:** 2018-04-16

**Authors:** Kyawt Mon Win, Jaya Prasad Tripathy, Thae Maung Maung, Tin Oo, Aung Thi, Khin Nan Lon, Zaw Lin

**Affiliations:** 1 Vector Borne Diseases Control Program, Ministry of Health and Sports, Naypyitaw, Myanmar; 2 International Union Against Tuberculosis and Lung Disease, The Union South-East Asia Regional Office, New Delhi, India; 3 Department of Medical Research, Ministry of Health and Sports, Yangon, Myanmar; 4 Yangon Regional Public Health Department, Department of Public Health, Ministry of Health and Sports, Yangon, Myanmar

**Keywords:** Lymphatic filariasis, Mass drug administration, Myanmar

## Abstract

**Background:**

As Myanmar progresses towards lymphatic filariasis (LF) elimination, it is important to know how well the anti-filarial activities have performed. The present study was conducted to study the implementation of the key anti-filarial activities and their impact on key indicators of LF transmission.

**Methods:**

A secondary analysis of aggregate program data on the anti-filarial activities was conducted in four endemic state/regions of Myanmar receiving at least six mass drug administration (MDA) rounds during 2001–2016.

**Results:**

MDA coverage has been expanded to cover all the endemic implementation units (IUs), i.e., 45 by 2015 and 6 IUs out of them have already stopped MDA. The reported coverage of MDA ranges from 87 to 100% whereas surveyed coverage ranges from 78 to 100% among the eligible population. The prevalence of microfilaria has significantly declined especially in Magway from 4.7 to 0.2% and Sagaing region from 7.9 to 1.3% during 2001–2016. Around 2.5% of estimated cases of hydrocele were reported to the program during 2009–2014.

**Conclusion:**

Myanmar has achieved significant success in interrupting LF transmission through several MDA rounds with high coverage. However, morbidity reporting and management, being in its initial phase requires an active surveillance system for identifying and managing people with LF-associated morbidities under the program.

## Background

Lymphatic filariasis (LF) is a debilitating neglected tropical disease currently infecting around 120 million people in 81 countries. An estimated 1.34 billion live in filarial endemic areas, with 65% of them residing in World Health Organization (WHO) South-East Asia Region (SEAR). About 40 million people suffer from the stigmatizing and disabling clinical manifestations of the disease, 15 million have lymphoedema (elephantiasis) and 25 million men have urogenital swelling, principally scrotal hydrocele [1].

LF is a major public health problem in the SEAR. Nine out of the 11 countries in the region are endemic for filariasis. In 2000, WHO established the Global Programme to Eliminate Lymphatic Filariasis (GPELF), to achieve LF elimination (LF antigenaemia < 1/1000 population) by 2020. To interrupt transmission, the WHO recommends an annual mass drug administration (MDA) of single doses of two medicines, namely diethylcarbamazine (DEC) or ivermectin plus albendazole to the entire eligible population in endemic areas for at least 5 years [2].

**Fig. 1:**
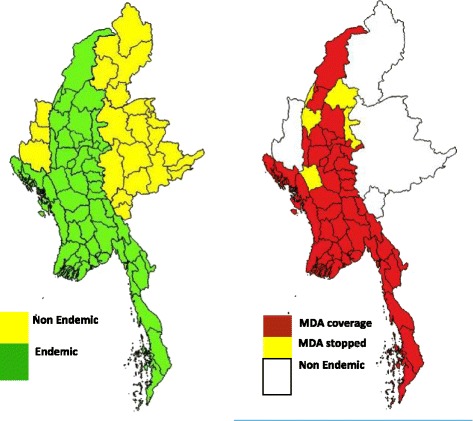
Map showing lymphatic filariasis-endemic and lymphatic filariasis-non-endemic regions and MDA coverage in Myanmar, 2016. MDA = mass drug administration

Myanmar is a LF endemic region covering 45/74 districts and 240/330 townships [3]. (Figure 1) Myanmar adopted the WHO guidelines as described above and developed the National Plan to Eliminate Lymphatic Filariasis (NPELF) in 2000 [1, 3].

Analyses of the effect of MDA on transmission of human infection have been documented in countries such as Egypt [4], Papua New Guinea [5, 6] American Samoa [7, 8], India [9], Sri Lanka [10], Thailand [11], Tanzania [12], and Nigeria [13]. However, there is no published literature on the effect of MDA on the control of filariasis in Myanmar. Among the Southeast Asian countries, Myanmar is considered to be one of the high-LF-burden countries. According to a recent review by Dickson et al., there is no scientific evidence from Myanmar on the burden of LF [14]. However, studies have reported high morbidity and LF prevalence along the Thai-Myanmar border and among the migrants from Myanmar in Thailand [15–17].

Myanmar has initiated MDA since 2001 and has expanded to cover the whole of the endemic region in the country with reportedly high coverage rates. This provides us the opportunity to study the effect of MDA on microfilarial transmission in the community under routine program settings. Success in eliminating filaria in Myanmar will require knowledge on how well the anti-filarial activities have succeeded so far.

Thus, the present study was conducted with the following specific objectives: (i) reporting the implementation of key anti-filarial activities (MDA administration and LF-associated morbidity case reporting) and (ii) studying the impact of these activities on key indicators like coverage of MDA, prevalence of microfilaria, and filarial antigenaemia in four filarial endemic state/regions (Magway, Sagaing, Rakhine, and Mandalay) during 2001–2016.

## Methods

### Study design

Aggregate data (ecological design) under the Vector Borne Disease Control (VBDC) Program were summarized at region and district/implementation unit (IU) level.

### General setting

Myanmar, situated in Southeast Asia, has an estimated population of around 52 million, of whom 70% live in rural areas [18]. Myanmar is 676,578 km^2^ in size with 49% forest cover. The country is divided administratively into the capital territory (Nay Pyi Taw Council Territory) and seven states and seven regions, and 74 districts with 412 townships/sub townships [18]. The wet climate and the mountainous topography favors LF transmission especially in the border areas. The districts vary in their population size ranging from 0.1–2.0 million.

### Study setting

**Fig. 2:**
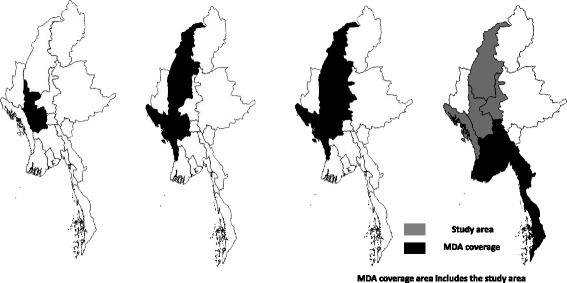
Map showing geographical expansion of MDA coverage in lymphatic filariasis regions in Myanmar during 2001–2016. MDA = mass drug administration

Under the LF elimination program in the country, the following activities have been carried out since 2000: area mapping of LF endemicity, MDA in LF endemic areas, evaluation of MDA (through MDA coverage surveys, night blood surveys, and Transmission Assessment Surveys), and morbidity control activities. In 2011, mapping for LF endemicity was completed which found that 45 out of 65 districts/IUs are LF endemic. MDA was started in 10 townships in Magwe region as a pilot project in 2001. Until 2016, MDA has covered all 45 endemic IUs Fig. 2.

**Fig. 3:**
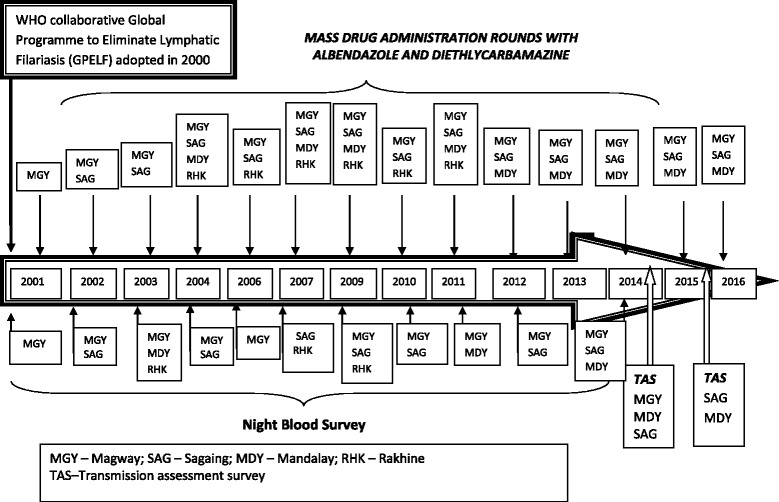
Chronological order of anti-filarial activities in four endemic regions under the Vector Borne Control Program, Myanmar, 2001–2016

Four rounds of post-MDA coverage surveys have been carried out to evaluate the reported coverage. Night blood surveys (in spot-check and sentinel sites) and transmission assessment surveys (TAS) were done in accordance with the guidelines. In night blood surveys, finger prick method was used to collect night blood smears in order to identify circulating LF parasite in the blood. Figure 3 shows the chronological order of all anti-filarial activities that have taken place under the LF control program from 2001 to 2015.

In each sentinel site, a sample was collected from at least 500 individuals aged over 5 years. These sites were chosen randomly from high transmission areas. In each IU/district, one sentinel site was selected. Once chosen, the same site remained as the sentinel site throughout the course of the program. However, different spot-check sites were chosen randomly for every assessment. At least one spot-check site was chosen per sentinel site.

MDA was started in 02 IUs of Magway region in 2001, later expanded to 04 IUs in 2002, and 14 rounds have been completed as of 2016. In Sagaing, MDA started in 06 IUs in 2002, and 13 rounds have been completed by 2016. Subsequently, MDA was stopped in 03 IUs in 2009. In Rakhine, MDA was initiated in 2004 in 04 IUs, which was subsequently stopped after having conducted 06 rounds. Similarly, in Mandalay, MDA started in 07 IUs in 2004; 09 rounds have been completed so far Table 1.

**Table 1:** Details of MDA coverage and compliance rates by in four endemic regions/states of Myanmar

Region/state	No. of endemic IUs	No. of MDA rounds	Period of MDA coverage	Reported coverage in % (range)	Compliance rate in % (range)
Magway	04^a^	14	2001–2016	95 (89–99)	94 (90–100)
Sagaing	06^b^	13	2002–2016	94 (87–100)	92 (78–100)
Mandalay	07	09	2004–2016	94 (89–97)	94 (93–95)
Rakhine	04^c^	06	2004–2011	96 (91–99)	96 (95–97)

*IU* implementation unit, *MDA* mass drug administration

^a^MDA started in 02 IUs in 2001, later 02 more IUs were added in 2002

^b^MDA started in 06 IUs, later stopped in 03 IUs in 2009

^c^MDA started in 04 IUs, later stopped in all 04 IUs in 2009

### Study population

The study population comprised of the general population in four endemic state and regions of Myanmar (Magway, Sagaing, Mandalay, and Rakhine) where at least six rounds of MDA have been conducted during 2001–2016.

### Study duration

Data compilation, cleaning, and verification were conducted between October 2016 and March 2017, using National VBDC Program data collected from the years 2001 to 2016**.** Data analysis and manuscript writing was done between April and June 2017.

### Data collection and variables

Aggregate data were collected from the Annual reports of VBDC Program, electronically maintained routine filariasis control program data and reports of surveys such as night blood survey, post-MDA coverage survey and transmission assessment survey during the period 2001–2016. These activities were done as part of the routine activities conducted to monitor and evaluate the anti-filarial control program. All these activities were done according to the standard WHO guidelines [1]. Operational definitions of key terms have been given in Annexure 1. The program data pertaining to LF morbidities (cases of hydrocele) were obtained from hospitals in the public sector. As the National Vector Borne Program staffs including the program manager were part of this study, any discrepancy in the data was cross checked with the respective region/state/township program staff.

### Analysis and statistics

Proportions were used to summarize key indicators such as reported and surveyed coverage of MDA, mf prevalence, and prevalence of filarial antigenaemia. Trends were presented in the form of a line diagram. Mf prevalence in sentinel and spot-check sites was classified into two categories based on a cut-off: < 1% and > = 1%. Subsequently, number of sites in each category was counted in each night blood survey and has been presented in a tabular form. Data validation was done by checking with the program staff at different levels. Maps were constructed using QGIS software (version 2.18.3) to denote the filarial endemic regions and the expansion of MDA coverage in the country. Estimated cases of hydrocele were calculated by taking prevalence of hydrocele as 5.9% among adults > 15 years of age from a community-based survey carried out in Mandalay region in 2014 (unpublished data). This was then projected onto the adult population (> 15 years) of the respective state/region. These estimated figures were then compared with the reported cases under the National VBDC Program.

### Ethics approval and consent to participate

Ethics approval for this study was obtained from The Union Ethics Advisory Group (International Union against Tuberculosis and Lung Disease, Paris, France) (EAG Number: 79/16) and the Institutional Ethics Review Committee, Department of Medical Research, Myanmar (Ethics/DMR/20117/018).

## Results

### Coverage of MDA rounds

MDA rounds started in 2 IUs of Magway in 2001 covering 1.8 million population expanding to 33 million in 2016. A total of 14 rounds of MDA have been conducted in Magway region, 13 rounds in Sagaing, and nine rounds in Mandalay whereas six rounds of MDA were carried out in Rakhine state within the period of 2001–2016. Table 1 shows the details of the number of endemic IUs, number of MDA rounds, and coverage of MDA in four study regions/states. MDA coverage has been expanded to cover all the endemic IUs (45) by 2015, and 6 IUs out of them have already stopped MDA in the study area Figs 1 and 2.

### Trend in prevalence of microfilaremia and MDA coverage

**Fig. 4:**
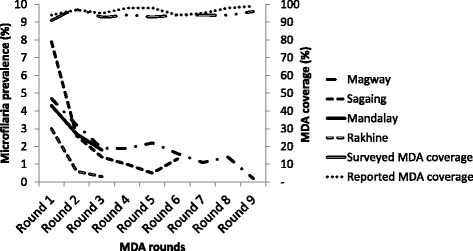
Trend in the prevalence of microfilaria and the overall MDA coverage after multiple MDA rounds in four endemic state/regions of Myanmar during 2001–2015. MDA = mass drug administration

The reported coverage of MDA rounds ranges from 87 to 100% for the eligible population whereas surveyed coverage ranges from 78 to 100% among the eligible population. The prevalence of microfilaria in Magway was 4.7% in 2001, which reduced to one-third (1.6%) in 2007 and further dipped to 0.2% in 2014. In Sagaing region, microfilaremia dipped from 7.9% in 2002 to 1.3% in 2014. Similarly, the Mandalay region and Rakhine state also witnessed a sharp decline in microfilaremia rates Fig. 4.

### Effect of MDA on mf prevalence in sentinel and spot-check sites

Tables 2 and 3 show the mf prevalence in sentinel and spot-check sites in several night blood surveys conducted during the study period in the four regions/states. It shows that the number of sentinel and spot-check sites with mf prevalence > = 1% have come down during this period. Table 4 shows the sentinel-wise trend in the prevalence of microfilaria. It suggests that although mf prevalence has come down significantly in many sites, some pockets in districts like Pakokku (Magway), Shwebo (Sagaing), Myingyan, Mandalay, Meikhtila, and Kyauk Se (Mandalay) have persistently high levels of mf prevalence. Table 5 shows the overall impact of MDA on mf prevalence in sentinel sites IU wise in four states/regions of Myanmar.

**Table 2:** Impact of MDA on microfilaria prevalence at sentinel sites in four endemic regions/states of Myanmar

Region/state	No. of endemic IUs^a^	Year of post-MDA survey	No. of sentinel sites	mf prevalence (range)	Overall mf prevalence (%)	No. of sentinel sites with mf prevalence < 1% > = 1%	
Magway	04	2001	04	1.18–7.1	4.7	0	4
		2002	04	0.19–9.17	3.9	1	3
		2003	04	0.19–4.0	2.6	1	3
		2004	04	0.0–2.5	1.45	1	3
		2005	04	0.4–3.4	1.8	1	3
		2007	04	0.0–2.5	1.1	2	2
		2008	04	0.0–2.8	1.12	3	1
		2010	04	0.0–2.7	1.45	2	2
		2011	04	0.0–0.4	0.2	4	0
		2012	02	0.8–2.2	1.5	1	1
		2014	01	0.33	0.33	1	0
Sagaing	06	2002	06	3.0–15.1	7.9	0	6
		2004	06	0.6–4.7	2.6	1	5
		2007	06	0.0–3.6	1.43	2	4
		2009	06	0.0–2.1	0.9	4	2
		2010	02	0.0–1.7	0.8	1	1
		2012	01	0.79	0.79	1	0
		2014	05	0.0–1.89	1.1	3	2
Mandalay	07	2003	10	0.2–14.7	5.0	3	7
		2011	13	0.0–8.8	2.7	5	8
		2014	12	0.0–3.2	1.19	6	6
Rakhine	04	2003	06	0.0–12.6	3.0	2	4
		2007	08	0.0–2.8	0.57	6	2
		2009	07	0.0–0.8	0.26	7	0

*IU* implementation unit, *MDA* mass drug administration, *mf* microfilaria

^a^In Magway, MDA started in 02 IUs in 2001, later 02 more IUs were added in 2002; In Sagaing MDA started in 06 IUs, later stopped in 03 IUs in 2009; In Rakhine, MDA started in 04 IUs, later stopped in all 04 IUs in 2009

**Table 3:** Impact of MDA on microfilaria prevalence at spot-check sites in four endemic regions/states of Myanmar

Region/state	No of endemic IUs	Year of post-MDA survey	No. of spot-check sites	mf prevalence (range)	Overall mf prevalence (%)	No. of sites with mf < 1% > = 1%	
Magway	04	2003	05	0.0–7.6	2.2	3	2
		2004	04	0.6–2.8	1.7	1	3
		2005	04	3.0–7.2	5.1	0	4
		2006	04	0.2–1.4	0.6	3	1
		2008	04	1.9–2.5	1.3	0	4
		2009	02	0.0–0.8	0.4	2	0
		2012	07	0.0–6.6	1.2	4	3
		2014	01	0.6	0.6	1	0
Sagaing	06	2004	06	0.0–6.6	2.6	4	2
		2007	06	0.0–5.6	2.7	1	5
		2008	04	0.2–5.6	2.0	2	2
		2009	06	0.0–5.6	1.5	4	2
		2014	05	0.0–0.0	0.0	5	0
Mandalay	07	2011	04	0.0–3.8	1.5	2	2
		2014	01	0.0	0.0	1	0
Rakhine	04	2008	04	0.0–0.6	0.3	4	0
		2010	04	0.0–0.6	0.2	4	0
		2015	02	0.0	0.0	2	0

*IU* implementation unit, *MDA* Mass Drug Administration, *mf* microfilaria

In Magway, MDA started in 02 IUs in 2001, later 02 more IUs were added in 2002

In Sagaing MDA started in 06 IUs, later stopped in 03 IUs in 2009

In Rakhine, MDA started in 04 IUs, later stopped in all 04 IUs in 2009

**Table 4:** Trend in the prevalence of microfilaria at different sentinel sites in four endemic region/states of Myanmar during the period 2001–2016

District	Township	Sentinel site	2001	2002	2003	2004	2005	2006	2007	2008	2009	2010	2011	2012	2013	2014
Magway																
Magway	Chauk	No 1 ward	7.1		3.6		1.7			2.8	1.0		0.4			
	Myothit	Myolulin	1.1		0.2		0.4			0.0			0.0			
Thayet	Thayet	PyiTawAye	6.3		2.4		3.4			0.8			0.4			
	Kanma	Pahtoe		3.0		1.2			0.0	0.9			0.0			
Pakokku	Pakokku	Kamma		0.2		0.0			0.0			0.0				
	Yesagyo	No. 8		9.2		2.5			2.5			2.7		2.2		
Minbu	Salin	Sinphyukyun		3.3		2.1			1.8			2.7		0.8		
	Minbu	KyaukTan	4.4		4.0		1.8					0.4				0.3
Sagaing																
Sagaing	Sagaing	Ayemyawaddy		7.0		3.0			3.6		0.9					
	Sagaing	Nyaungpinwynn		3.0		1.0			0.6		0.2					
Monywa	Tabayin	Aye tharyar		5.5		2.2			1.6		0.4					
	Budalin	Minywa		5.2		0.6			0.0		0.0					
Shwebo	Taze	PawOo, BoMya		15.1		4.7			1.2		2.1	1.7		0.8		1.9
	Depeyin	Saipyin		11.5		3.9			1.6		1.8	0.0				1.3
Mandalay																
Nyaung U	Nyaung U	Tharyarwady											0.4			1.3
	Nyaung U	Taung Ba											0			
Yamethin	Leway	Naung Bo			3.0								2.5			0.5
	Tatkone	Sayasan ward			6.0								5.2			0.2
Meikhtila	Thazi	Nyaungyan			1.7								0.2			0.2
	Wandwin	Myopaw Ward			7.2								4.6			1.9
Mandalay	Amarapur	Tharlayswa			6.8								8.8			1.7
	Maha aung myay	Thanhletmaw west			0.7								0.6			0.2
Kyauk Se	Tada Oo	Thargaung											3.7			2.1
	Myitha	Ahshe/Taung ywa											1.5			
Myingyan	Kyaukpadaung	Daungle			13.3								4.6			3.2
	Nahtogyi	Tanzin			3.4								2.7			1.3
Pyin Oo Lwin	Madaya	Latkaunglay			0.2								0.0			
	Pyin Oo Lwin	Myopaw ward			0.4											0.3
Rakhine																
Sittwe	Sittwe	Kathe			12.6				2.8		0.8					
	Sittwe	Shanywa			1.4				0		0					
Maungdaw	Maungdaw	Kyainchaung			0.16				0		0					
	Maungdaw	No.1 ward			1.7				0		0					
Kyaukphyu	Pauktaw	Paikseik			2.34				1		0.2					
	Kyaukphyu	T.b.chaung							0.8		0.8					
Thandwe	Thandwe	Kinmaw			0				0		0					
	Thandwe	No.1 ward							0							

Figures in the tables are microfilaria prevalence expressed in percentages

**Table 5:** Impact of MDA on mf prevalence at the level of the implementation unit in four states/regions of Myanmar during 2001–2016

State/region	Name of IU	Baseline mf prevalence	No of MDAs	MDA compliance rate (range)	Mf prevalence (range)	No. of sites with mf prevalence		Filarial antigen prevalence	Decision about MDA status
						< 1%	> = 1		
Magway	Magway	4.1	13	92–96	0.0–7.1	5	6	2.4	Continue
	Thayet	7.1	13	93–96	0.0–6.3	5	5	3.6	Continue
	Pakoku	9.2	13	92–96	0.0–9.2	4	5	3.8	Continue
	Minbu	4.4	12	94–97	0.3–4.4	3	7	3.3	Continue
Sagaing	Sagaing	5	12	93–96	0.2–7	3	5	4.7	Continue
	Monywa	5	12	92–96	0.0–5.5	4	4	3.8	Continue
	Shwebo	8	12	93–95	0.0–15.1	2	11	6.8	Continue
Mandalay	Nyaung U		8	94–97	0.4–1.3	2	1	0.8	Continue
	Yamethin	4.5	8	93–97	0.2–6.0	2	4	2.4	Continue
	Meikhtila	4	8	92–95	0.2–7.2	2	4	2.6	Continue
	Mandalay	4.5	8	91–96	0.2–8.8	3	3	3	Continue
	Kyauk Se		8	94–96	1.5–3.7	0	3	2.4	Continue
	Myingyan	8	8	93–97	1.3–13.3	0	6	3.2	Continue
	Pyin Oo Lwin	0.3	8	92–97	0.0–0.4	5	0	0.5	Stop
Rakhine	Sittwe	7	6	92–96	0.0- 12.6	3	3	1	Stop
	Maungtaw	1	6	93–96	0.0–1.6	5	1	0.8	Stop
	Kyaukphyu	1	6	94–97	0.0–2.34	3	2	1	Stop
	Thandwe	0	6	95–96	0–0	4	0	0	Stop

### Reporting of hydrocele cases

**Fig. 5:**
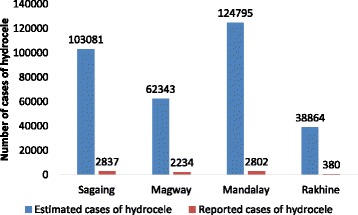
Estimated cases versus reported cases of hydrocele in four endemic state/regions of Myanmar, 2009–14

Figure 5 shows the estimated cases versus reported cases of hydrocele in four endemic state and regions of Myanmar during 2009–2014. Around 2.5% of estimated cases of hydrocele were reported to the program.

### Results of transmission assessment surveys

TAS has been conducted in 07 out of 21 IUs in the four endemic state/regions. Overall, 14,476 children were tested for LF antigen in TAS and 36 (0.25%) had test positive. TAS in three state/regions (6 out of 07 IUs) showed very low proportion (< 1%) of LF antigenaemia leading to stoppage of MDA rounds in those regions. The LF antigenaemia in Shwebo district of Sagaing region was 1.4% leading to continuation of MDA Table 6.

**Table 6:** Transmission Assessment Survey in four endemic state/regions of Myanmar, 2008–2015

Region	District/IU	TAS	Year	Children (*N*)	Test positive *N* (%)	MDA status
Sagaing	Kalay	Yes	2008	3011	0(0.0)	Stopped
	Tamu	Yes		3081	0(0.0)	Stopped
	Kathar	Yes		3003	0(0.0)	Stopped
	Shwebo	Yes	2015	2104	30(1.4)	Continue
	Sagaing	No				
	Monywa	No				
Magway	Minbu	Yes	2014	1565	1(0.06)	Stopped
	Pakokku	No				
	Magway	No				
	Thayet	No				
Mandalay	Pyin Oo Lwin	Yes	2014	2500	0(0.0)	Stopped
	Nyaung Oo	Yes	2015	1712	5(0.3)	Stopped
	Kyauksel	No				
	Myingyan	No				
	Yae Mae Thin	No				
	Mandalay	No				
	Meikhti Lar	No				
Rakhine	Maungdaw	No				
	Mrauk U	No				
	Kyauk Pyu	No				
	Thandwe	No				
Overall				14,476	36(0.25)	

*MDA* mass drug administration, *NA* not applicable, *TAS* Transmission Assessment Survey, *IU* implementation unit

## Discussion

This is one of the very few studies in Myanmar showing the impact of various anti-filarial activities on key program indicators since the country adopted the WHO GPELF. The two key findings of the study are (a) at least six MDA rounds with high coverage rates in the endemic regions have led to a significant decline in the prevalence of microfilaria and filarial antigenaemia leading to stoppage of MDA in six implementation units, and (b) there is gross underreporting of cases of hydrocele, which is one of the significant morbidities associated with LF.

This evidence from Myanmar adds to the growing body of literature demonstrating that five to six rounds of MDA with high coverage, using DEC+ALB, will reduce mf prevalence to < 1.0%, hence having significant implications for LF elimination programs [9, 13, 19]. However, this interpretation should be read with caution because the present study reports only MDA coverage, i.e., receipt of drugs whereas GPELF actually recommends a threshold of 65% compliance, i.e., actual ingestion of tablets for LF elimination. Although there are post-MDA surveys reporting actual MDA coverage, there are no reliable data on the actual compliance to MDA due to lack of clear definitions. This has important implications for setting and reaching elimination targets. A review of 36 MDA studies by Babu et al. in India found compliance to be 22% lower than MDA coverage [20]. Another review by Shuford et al. in 2016 revealed substantial heterogeneity across terminologies and definitions used to assess compliance [21]. Standardization of the compliance definitions coupled with longitudinal research in systematic non-compliance should be essential elements in the programmatic shift from control to elimination. Compliance surveys following each MDA round should be done under supervision and with the help of trained investigators and using a standard procedure to get more reliable data on actual consumption of drugs. Directly observed swallowing of the tablets during MDA rounds should be emphasized and monitored.

In the four endemic state/regions with more than six MDA rounds, only seven TAS have been conducted out of 21 IUs eligible for TAS. TAS provides the evidence for stoppage of MDA and thus is an important step towards LF elimination. The country should make all efforts to conduct further TAS rounds in accordance with the NPELF. Support from international agencies is required in this endeavor.

Although night blood surveys have shown decrease in the number of sentinel/spot-check sites with mf prevalence > = 1%, certain pockets (sentinel sites) have shown persistence of microfilaria which requires intensification of the LF activities and regular monitoring. Despite several rounds of MDA, pockets of high microfilaremia might indicate poor compliance to the drugs in terms of actual ingestion of the drugs, which needs further investigation.

Entomological surveillance is another method for monitoring the impact of MDA on LF transmission, apart from serological and parasitological indicators [1]. However, review of anti-filarial activities in the last 16 years in Myanmar showed no such activity. As we progress towards LF elimination, this could be a key indicator in monitoring our progress towards the goal of elimination of LF, thus warranting its inclusion in the VBCD Program strategy [1, 22].

Managing morbidity and preventing disability among those already affected by LF is one of the two main pillars of LF elimination. To this end, the GPELF recommends that all endemic countries should be collecting and reporting data on morbidity management, although there is no recommended method for collecting such data [1]. Currently, the focus of VBDC in Myanmar is primarily on interrupting transmission through MDA. The present study reports gross underreporting of hydrocele cases. Moreover, the miniscule number of cases which are reported are in fact hospital-reported ones, and that too from public sector hospitals only. This means that many undiagnosed cases of hydrocele in the community go unreported. Also, there is no data on other morbidities such as lymphedema. There is no mechanism of active community-based case reporting of LF-associated morbidities such as hydrocele and lymphedema. Global efforts in terms of morbidity prevention are yet to gain momentum with only 27 out of 81 endemic countries having active morbidity management programs. It is thus strongly recommended that an active community-based surveillance of cases and a plan to manage people with such morbidities should be put in place under the VBDC Program in Myanmar [23]. Hospital-based reporting of LF morbidities should be strengthened. The program also must focus on managing chronic morbidities, which persists even after transmission has been interrupted. This requires strengthening of the health facilities in delivering minimum package of MMDP (Morbidity Management and Disability Prevention) care.

What are the key challenges to LF program implementation in Myanmar? We speculate that decades of political and economic turmoil, unique geography, and migration dynamics have played their part in the continued transmission of the disease. Lack of reliable epidemiological data and a constrained public health system are also retarding efforts to eradicate the disease. Although the WHO recommends annual treatment, there have been gaps of 1–2 years between rounds as a result of financial and logistic issues. Despite these challenges, Myanmar has continued to make significant progress towards LF elimination. However, it requires sustained funding and a robust surveillance system yielding accurate data.

The major strength of the study is that it reported program data from four major LF endemic states/regions during a 16-year period (2001–2016) since Myanmar formulated its NPELF in 2000. This is also the first piece of evidence from Myanmar documenting the impact of several rounds of MDA on LF transmission. We feel that the results of the study could be generalizable to the entire country because of similar program guidelines, demography and other factors affecting LF transmission. However, the aggregate data analyzed in this study were obtained from the routine program records, and there was no means of validating the data which we acknowledge as a limitation in this study.

## Conclusion

In four high-LF endemic regions/states, Myanmar has done well in interrupting LF transmission through several MDA rounds with high coverage, but morbidity reporting and management has not been given due attention. Thus, an active surveillance system for identifying and managing people with LF-associated morbidities should be instituted under the program. The program should also make efforts to get reliable epidemiological data for continuous monitoring and evaluation.

## Abbreviations

DEC: Diethylcarbamazine
GPELF: Global Programme to Eliminate Lymphatic Filariasis
IU: Implementation unit
LF: Lymphatic filariasis
MDA: Mass drug administration
NPELF: National Plan to Eliminate Lymphatic Filariasis
TAS: Transmission Assessment Survey
VBDC: Vector Borne Disease Control
WHO: World Health Organization
